# Profile of Patients with Maxillofacial Space Infections and Associated Risk Factors

**DOI:** 10.1155/2024/9304671

**Published:** 2024-04-09

**Authors:** Srikanth Gadicherla, Kirti Manglani, Kalyana C. Pentapati, Adarsh Kudva, Chithra Aramanadka, Rajaji Chandravel

**Affiliations:** ^1^Department of Oral and Maxillofacial Surgery, Manipal College of Dental Sciences, Manipal Academy of Higher Education (MAHE), Manipal, Karnataka, India; ^2^Department of Public Health Dentistry, Manipal College of Dental Sciences, Manipal Academy of Higher Education (MAHE), Manipal, Karnataka, India; ^3^Health Services Management, University of Chester, Chester, UK

## Abstract

**Objective:**

To evaluate the profile of patients operated for maxillofacial space infections and associated risk factors for the length of hospital stay.

**Materials and Methods:**

We conducted a retrospective study among patients operated for maxillofacial infections at our center from 2010 to 2020. Information collected from the records were age, sex, type and number of spaces involved, clinical signs and symptoms (pain, swelling, toothache, sore throat, otalgia, hoarseness, headache, cough, neck swelling, rancid breath, sialorrhea, gingival swelling, muffled voice, trismus, fever, dysphagia, odynophagia, malaise, lymphadenopathy, dyspnoea, pus discharge), treatment modality, total leukocyte count, evidence of bacterial growth, comorbidities, complications if any and length of hospital stay.

**Results:**

A total of 128 medical records were examined, out of which 59 were female. The mean age was 38.59 ± 19.7 and the length of hospital stay was 7.56 ± 3.8 days. The most commonly involved space was submandibular space (46.1%) and the common symptoms reported were swelling (99.2%), pain (86.7%), and trismus (68%). Four patients had complications like necrotizing fasciitis (1.6%), pneumonia (0.8%), and death in one patient (0.8%). Logistic regression showed that patients more than 36 years of age, male sex, evidence of bacterial growth, and diabetics had higher odds of increased hospital stay (>6 days). Multiple logistic regression analysis showed that age (*P* = 0.015; OR: 2.98) and evidence of bacterial culture (*P* = 0.001; OR:6.64) were potential predictors associated with increased hospital stay.

**Conclusion:**

Our study showed that the age of the patient and evidence of bacterial culture were potential predictors of prolonged hospital stay among patients operated for maxillofacial space infections.

## 1. Introduction

Maxillofacial space infections (MFSI) are a group of potentially serious infections commonly encountered and leading causes of potentially preventable hospitalizations [1] by oral and maxillofacial surgeons. They are characterized by the spread of bacterial infections from the teeth or supporting structures into adjacent spaces in the head and neck region. These infections typically result from untreated dental caries, periodontal disease, dental trauma, or surgical complications, allowing bacteria to invade deeper tissues through pathways such as blood vessels or fascial planes [2]. Most of the dental space infections are generally odontogenic origin [3].

The commonly affected spaces are the submandibular, sublingual, buccal, pterygomandibular, and submental spaces. These can extend to deeper spaces, leading to potentially life-threatening complications that include cellulitis, necrotizing fasciitis, cavernous sinus thrombosis, pneumonia, septicemia, airway obstruction, or even death [4]. Signs and symptoms include pain, swelling, difficulty in swallowing, fever, malaise, trismus, pus discharge, and lymphadenopathy. Prompt diagnosis and immediate treatment are vital to prevent the spread of infection, decrease hospital stay, and decrease the risk of complications. The management typically requires hospitalization, incision, and drainage of the infected space, and removal of the focus of infection with antibiotic therapy [2, 5].

Owing to the advances in dental care and the availability of antibiotics, there is a downward trend in the incidence of MFSI. However, they pose a significant public health concern due to their potential to spread rapidly which can cause complications. Hence, oral health care professionals must have thorough knowledge about the clinical presentation of MFSI, timely diagnosis, and appropriate treatment to prevent such life-threatening complications.

Previous studies on MFSI have reported various risk factors and profiles of patients [4, 6–13]. Literature on the association predictors of length of hospital stay in MFSI is scant [7, 14–16].

Studies have shown that elderly people [7, 11, 14], diabetes [7, 14–16], prior antibiotic usage [14], elevated leukocyte count [11], number of spaces involved [17], odontogenic infection severity score [14], changes in atmospheric pressure [18], and severity of infection [17] are significant predictors of prolonged hospital stay.

MFSI may be similar among patients in various geographic regions, but a thorough understanding of the prevailing risk factors specific to a community is required for careful planning and management of MFSI. This can prevent complications and decrease the length of hospital stay, which will eventually reduce the overall burden on the individual, community, and health care professionals. Moreover, the average length of hospital stay varies with different geographic locations and hence the magnitude of the associated risk factors varies. Against this background, we aimed to evaluate the profile of patients operated for MFSI and identify the potential risk factors that may influence the length of hospital stay.

## 2. Materials and Methods

We conducted a retrospective study among patients with space infections in the maxillofacial region in the oral and maxillofacial surgery department. The records of the patients between January 1^st^, 2010, to December 31^st^, 2020, were retrieved from the archives by a trained and calibrated examiner. The protocol was approved by the ethics committee of Kasturba Hospital and Kasturba Medical College (IEC: 61/2022). All patients were diagnosed based on the clinical findings and a standard treatment protocol was followed.

We included only patients who operated in the oral and maxillofacial surgery department for space infections. We excluded patients with incomplete records, operated elsewhere and referred to our center space infections secondary to trauma or road traffic accidents, or some other surgery.

Information collected from the records were age, sex, type and number of spaces involved, clinical signs and symptoms (pain, swelling, toothache, sore throat, otalgia, hoarseness, headache, cough, neck swelling, rancid breath, sialorrhea, gingival swelling, muffled voice, trismus, fever, dysphagia, odynophagia, malaise, lymphadenopathy, dyspnoea, pus discharge), treatment modality, total leukocyte count, evidence of bacterial growth, comorbidities, complications if any (cellulitis, necrotizing fasciitis, postdrainage trismus, further spread of infection, trigeminal nerve deficit, septicemia, pneumonia, airway obstruction, and death), length of hospital stay.

### 2.1. Statistical Analysis

All analysis was done using SPSS version 20. A *P* value of <0.05 was considered statistically significant. The median split method was used to categorize age and length of hospital stay. Bivariate analysis was performed using the Chi-square test between the length of hospital stay and various predictors. Logistic regression was performed to evaluate the association of significant predictors with outcome.

## 3. Results

**Figure 1:**
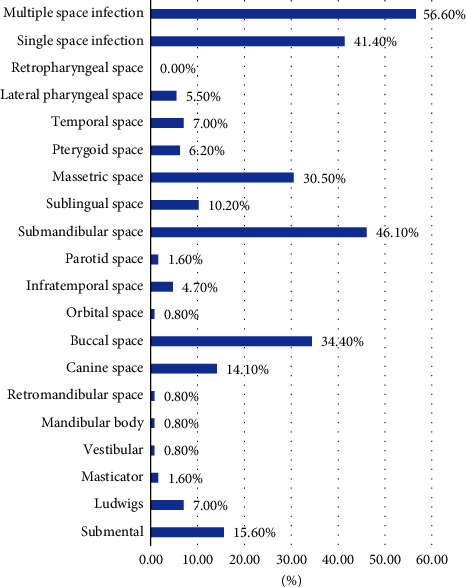
Distribution of space infections in the maxillofacial region.

**Figure 2:**
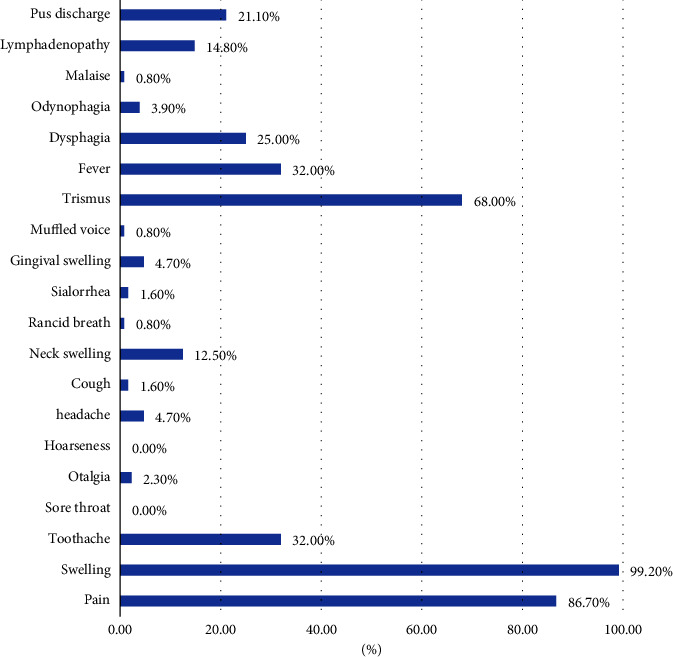
Distribution of signs and symptoms among patients with maxillofacial space infections.

A total of 128 medical records were examined, out of which 59 were female. The mean age of the patients was 38.59 ± 19.7 (Median: 36). The most commonly involved space was submandibular space (46.1%), followed by the buccal space (34.4%) and masseteric space (30.5%) (Figure 1). The most common symptoms reported were swelling (99.2%), pain (86.7%), and trismus (68%) (Figure 2). A total of 17.2 and 16.4% of patients were hypertensive and diabetic under medication (Table 1). Almost all patients were treated using incision and drainage and most of them required drain. Four patients had complications like necrotizing fasciitis (1.6%), pneumonia (0.8%), and death in one patient (0.8%). More than half of the patients had multiple space infections (58.6%), one-third of them had fever (32%), more than one-quarter of the patients had elevated total leukocyte counts (28.6%) and more than 1/5th of the patients had bacterial growth. The mean hospital stay was 7.56 ± 3.8 days (median = 6).

Bivariate analysis was performed to evaluate the role of predictors on the length of hospital stay. It was seen that patients more than 36 years of age, males, with evidence of bacterial growth, and diabetic individuals had higher odds of increased hospital stay of more than six days (Table 2). Multiple logistic regression was used to evaluate the association of the factors that were significant in the binomial logistic regression. It was seen that only age (*P*=0.015; OR: 2.98; 95% CI: 1.24–7.18) and evidence of bacterial culture (*P*=0.001; OR:6.64; 95%CI: 2.2–19.97) were the potential risk factors associated with an increased hospital stay of more than 6 days.

## 4. Discussion

In this retrospective study, we examined the association between length of hospital stay and predictors among patients operated on for MFSI. To the best of our knowledge, this is the first study that evaluated the association between length of hospital stay and predictors of MFSI in the Indian population. Sex distribution among MFSI patients was similar to that in earlier studies [7, 14–16, 19]. The mean age of the patient was 38 years, which was similar to Gams et al. [14] but lower than Park et al. [7], Zawiślak and Nowak [20], and higher than Peters et al. [16]. More than half of the patients had involvement in multiple spaces, which was similar to Zhang et al. [9].

The mean length of hospital stay in our study was 7.6 days which was similar to Storoe et al. (6.6 and 8.27 days) [21]. This was higher than Tan et al. (3 days) [19], Kamisnki et al. (5 days) [22], Gams et al. (5.46 days) [14], Peters et al. (4.7 days) [16], Kim et al. (3.9 days) [15], Allareddy et al. (3.9 days) [23] but lower than Park et al. (12.43 days) [7]. Our study used the median (6 days) to define short or prolonged length of hospital stay. Previous studies have used a variety of criteria to define prolonged hospital stay which varies substantially in different geographic locations. This may be due to prevailing treatment guidelines, availability of insurance, and other resources. It is noteworthy to mention that changes in reimbursement through insurance have changed the management strategies of less severe MFSI from elective surgery to outpatient services.

Our study showed that only age (OR: 2.98) and evidence of bacterial culture (OR:6.64) were the potential risk factors associated with increased length of hospital stay (>6 days). Park et al. showed that elderly patients more than 65 years of age and diabetes are potential predictors among patients presenting to emergency wards [7]. Gams et al. reported that predictors like age, prior antibiotic usage, diabetes, and odontogenic infection severity score were significantly associated with length of hospital stay among patients with severe odontogenic infections [14]. Kim et al. reported that uncomplicated diabetes was significantly associated with length of hospital stay among patients with cellulitis [15]. Wang et al. reported that age and elevated leukocyte count are significant predictors of prolonged hospital stay among patients with odontogenic infections [11]. Peters et al. reported that preexisting medical conditions that are immunosuppressive are significantly associated with length of hospital stay among patients with maxillofacial space infections [16]. However, Kaminski et al. reported that the duration of hospital stay was marginally higher among diabetics than among nondiabetics [22]. Flynn et al. reported an association between the severity of the infection and the number of spaces involved spaces among patients with severe odontogenic infections [17]. Tan et al. [19] reported that increased length of hospital stay was associated with age and physical status classification. Rasteniene et al. indicated that longer period of hospitalization was associated with anaerobic bacteria, multiple space involvement, and odontogenic etiology among children and adolescents [24].

Increased hospital stay was shown to utilize substantial resources which can be a burden to health care professionals, individuals, and the overall community. Hence, knowledge about the potential predictors of increased hospital stay would help clinicians identify high-risk patients and initiate treatment protocols at the earliest. This will minimize complications and improve the outcomes among the patients. There is a need to implement educational programs for the public and medical fraternity regarding the potential outcomes of odontogenic infections. Efforts should be made to diagnose and initiate appropriate antibiotics at an early stage. This will reduce morbidity and may reduce the hospitalization rate and decrease the length of hospital stay.

The present study was retrospective and includes data from only one tertiary care center. Although we can demonstrate the association, we cannot establish the causation due to the cross-sectional study-design. We have included only patients who had odontogenic infections in the maxillofacial region and were operated at our center.

## Figures and Tables

**Table 1:** Distribution of comorbidities among patients with maxillofacial space infections.

	*N*	%
Diabetes	21	16.4
Hypertension	22	17.2
Others		
Anaemia	1	0.8
Asthma	5	3.9
Cardiovascular disease	4	3.1
Hypothyroid	4	3.1
Infectious disease	3	2.3
Mental illness/seizures	9	7.0
Pregnant/Lactating mother	4	3.1

**Table 2:** Bivariate analysis between length of hospital stay and various predictors.

	Length of hospital stay		*P* value	OR (95% CI)
	≤6 days	More than 6 days		
	*N* (%)	*N* (%)		
Age in years				
≤36	45 (65.2)	20 (33.9)	<0.001	3.7 (1.76–7.6)
>36	24 (34.8)	39 (66.1)		
Sex				
Female	38 (55.1)	21 (35.6)	0.028	2.22 (1.09–4.53)
Male	31 (44.9)	38 (64.4)		
Total leukocyte count				
<15000	52 (77.6)	38 (64.4)	0.102	1.92 (0.88–4.19)
>15000	15 (22.4)	21 (35.6)		
Number of spaces involved				
Single	32 (46.4)	21 (35.6)	0.217	1.57 (0.77–3.19)
Multiple	37 (53.6)	38 (64.4)		
Bacterial growth				
No growth	64 (92.8)	38 (64.4)	<0.001	7.07 (2.46–20.31)
Growth present	5 (7.2)	21 (35.6)		
Fever				
No	44 (63.8)	43 (72.9)	0.271	0.66 (0.31–1.39)
Yes	25 (36.2)	16 (27.1)		
Diabetes				
No	62 (89.9)	45 (76.3)	0.039	2.76 (1.03–7.38)
Yes	7 (10.1)	14 (23.7)		

OR: odds ratio; CI: confidence interval.

## Data Availability

Data are available upon request from the corresponding author.
